# Fall Risk and Knowledge of Fall-Risk-Increasing Drugs Among Saudi Older Adults

**DOI:** 10.3390/healthcare13131549

**Published:** 2025-06-29

**Authors:** Ayesha Yasmeen, Mamoon H. Syed, Abdulkarim M. Meraya, Saad S. Alqahtani, Nabeel Kashan Syed, Aseel J. Alfaifi, Mujeeb Alrhman I. Madkoor, Hilal A. Thaibah, Amani Khardali, Marie Claire Van Hout

**Affiliations:** 1 Department of Clinical Practice, College of Pharmacy, Jazan University, P.O. Box 114, Jazan 45124, Saudi Arabia; amoinuddin@jazanu.edu.sa (A.Y.); ameraya@jazanu.edu.sa (A.M.M.); nabeelsyed2020@outlook.com (N.K.S.); hthaibah@jazanu.edu.sa (H.A.T.); aakherdeli@jazanu.edu.sa (A.K.); 2 Pharmacy Practice Research Unit, College of Pharmacy, Jazan University, P.O. Box 114, Jazan 45124, Saudi Arabia; 3 Department of Clinical Pharmacy, College of Pharmacy, King Khalid University, P.O. Box 960, Abha 61421, Saudi Arabia; ss.alqahtani@kku.edu.sa; 4 Clinical Pharmacy Services, King Saud University Medical City, King Saud University, Riyadh 12372, Saudi Arabia; aalfaifi4@ksu.edu.sa; 5 Allied Health Services, Jazan Health Cluster, Jazan 82721, Saudi Arabia; mmadkoor@moh.gov.sa; 6 Office of the Vice President for Research, Innovation and Impact, South East Technological University, X91 AK3T Waterford, Ireland; mcvanhout@wit.ie

**Keywords:** fall risk, older adults, STEADI tool, pharmacist counseling, Saudi Arabia

## Abstract

**Introduction:** Falls pose a significant health risk to older adults, with a reported prevalence of 31.6% among Saudi older adults. Medication-related falls are a preventable cause of morbidity and mortality. This study aimed to assess fall risk, evaluate knowledge of fall-risk-increasing drugs, and examine the impact of pharmacist counseling on community-dwelling older adults in Jazan, Saudi Arabia. **Methods:** A cross-sectional survey was conducted from December 2023 to March 2024 among 391 community-dwelling individuals aged ≥60 years in Jazan, Saudi Arabia. Fall risk was assessed using the Arabic Stay Independent screening tool, which remains unvalidated in Arabic-speaking populations. Participants answered demographic questions and reported any pharmacist counseling on medication in the past six months. Knowledge of prescription and over-the-counter fall-risk-increasing drugs was evaluated. Multivariable logistic regression and ordered probit models were used to analyze factors associated with fall risk and drug knowledge. **Results:** Approximately 57% of the participants were at risk of falling. Only 11.5% demonstrated good knowledge of prescription fall-risk-increasing drugs, whereas 24.6% showed good knowledge of over-the-counter fall-risk-increasing drugs. Age (OR, 1.07; 95% CI, 1.00–1.14; *p* = 0.05), arthritis (OR, 5.73; 95% CI, 2.51–13.06; *p* < 0.001), obesity (OR, 6.00; 95% CI, 2.33–15.46; *p* < 0.001) and diabetes (OR, 2.79; 95% CI, 1.38–5.64; *p* = 0.004) were associated with increased fall risk. Those who received pharmacist counseling had a greater likelihood (95% CI, 0.020–0.167; *p* = 0.01) of being in the very likely category of willingness to discuss medication changes. **Conclusions:** The findings highlight the role of pharmacist counseling and recommend improving fall prevention through medication reviews for arthritis and diabetes patients, standardized counseling protocols, and implementation of the Stay Independent screening tool for risk assessment in older adults.

## 1. Introduction

Falls pose a significant health risk and are the leading cause of injuries and fatalities related to such accidents in older adults [1]. According to the United Nations, an older person is defined as an individual aged 60 years or older [2]. More than 30% of community-dwelling older adults experience falls annually, which result in disability and premature mortality [3]. In addition to physical injury, falls precipitate a fear of falling, leading to restriction in activity, limitation in mobility, increased possibility of subsequent falls, adverse effects on self-confidence, and decline in quality of life [4]. Globally, falls have an estimated prevalence of 26.5% [5]. Falls represent a significant challenge in geriatric care, often termed one of the “Geriatric Giants”. They are a leading cause of morbidity and mortality among older adults, with serious implications for public health., Nevertheless, falls are among the most frequently documented nursing diagnoses in hospitalized populations [6], reflecting their relevance as a cross-setting indicator of clinical and nursing care complexity—especially in adult and pediatric patients with multiple comorbidities or functional impairments. In recent decades, the prevalence of falls has been reported to be 47% among older adults in Gulf Cooperation Council (GCC) countries, including Saudi Arabia, which may be attributed to their higher life expectancies [7]. An observational study using Global Burden of Disease Data tracked falls in Saudi Arabia from 1990 to 2019, noting a rise in prevalence of 57% for males and 26% for females [8].

Fall-risk-increasing drugs (FRIDs) contribute to falls through multiple physiological pathways [9,10,11,12,13]. Psychotropic medications—such as benzodiazepines, antidepressants, and antipsychotics—act on the central nervous system and may cause sedation, dizziness, impaired coordination, and cognitive slowing [9,10]. These effects can increase postural instability and disrupt gait, particularly among older adults with pre-existing balance limitations. Cardiovascular drugs, including alpha-blockers, beta-blockers, vasodilators, and diuretics, can cause orthostatic hypotension or bradycardia, both of which may lead to syncope or imbalance [11]. Diuretics may also reduce serum sodium and potassium levels, further exacerbating neuromuscular dysfunction [11]. Opioids increase fall risk through combined mechanisms of sedation, delayed reaction time, and postural hypotension [12]. Moreover, polypharmacy and the concurrent use of multiple FRIDs are independently associated with higher fall risk due to synergistic adverse effects and drug–drug interactions [12,13].

Despite the well-established risks of these medications, awareness among older adults remains limited. Studies have shown that many older individuals are unable to identify common fall-risk-increasing drugs, particularly those available over the counter [14]. This lack of awareness is especially problematic in populations where polypharmacy is prevalent, and self-medication is common. Medications like diphenhydramine and certain antihistamines, frequently used for sleep or allergies, are often perceived as harmless despite their known sedative and anticholinergic effects [15]. These misconceptions contribute to preventable fall incidents and underscore the need to assess medication knowledge as part of broader fall prevention strategies.

The Jazan region of Saudi Arabia is among the smallest provinces of the country, with a population of approximately 1.4 million, and has the highest population density [16,17]. A 2020 cross-sectional study reported a mean age of 53.7 years among patients with chronic non-communicable diseases, suggesting rising healthcare demands among older residents [18]. However, disparities in healthcare access persist—17% of patients with chronic illnesses reported missed follow-up visits, with older adults, women, rural residents, and those with lower literacy being disproportionately affected [18,19]. Primary healthcare centers (PHCs), especially in rural areas, are often under-resourced, lack specialist services, and do not operate 24/7 [19]. Pharmaceutical care is primarily provided through these PHCs by general and family medicine physicians. Meanwhile, the widespread use of over-the-counter medications underscores the need for improved education and pharmacist involvement in medication safety, particularly among older adults managing chronic conditions [20].

Recent systematic reviews further highlight the complexity of medication-related fall risks. A 2022 review identified polypharmacy, older age, lower education, and comorbidities as significant risk factors for falls, while also noting that urban residence and living alone amplify this risk—both highly relevant to the healthcare landscape in Jazan [21]. A 2023 systematic review conducted in Saudi Arabia found the prevalence of potentially inappropriate medications (PIMs) among older adults ranged from 19% to 80%, with commonly implicated drugs including diuretics, antidepressants, and NSAIDs [22]. These findings are particularly concerning given the dominance of primary care settings and limited specialized pharmaceutical oversight outside tertiary centers in regions like Jazan. Another review, published in 2022, emphasized that while medication reviews alone are insufficient, they are a critical component of multifactorial fall prevention programs, especially when integrated with patient education and pharmacist-led interventions [23]. However, the direct impact of pharmacist-led interventions on fall rates remains unclear. A randomized STEADI-Rx trial found no significant reduction in fall risk versus usual care, highlighting the need for multifactorial approaches [24]. Although, there is a lack of specific data pertaining to the Jazan region, barriers in the Southern region include limited pharmacist training, lack of screening tool use (e.g., Beers, STOPP/START), and insufficient collaboration [25]. While pharmacist-led interventions effectively optimize medication regimens, their impact on fall incidence depends on integration with broader fall prevention strategies.

**Figure 1:**
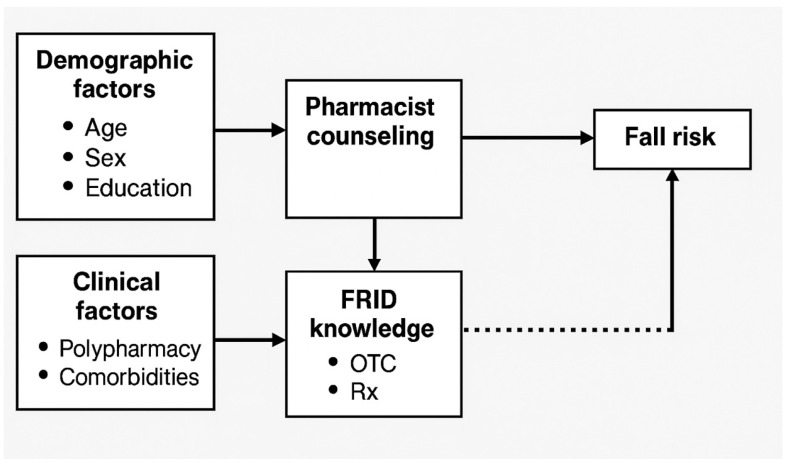
Conceptual model.

This study is grounded in an empirical framework that considers fall risk among older adults to be influenced by a combination of demographic, clinical, and behavioral factors. Among the behavioral factors, awareness of fall-risk-increasing drugs (FRIDs)—both prescription and over-the-counter—plays a critical role in shaping fall prevention behaviors. Pharmacist counseling is positioned as a modifiable factor that can enhance medication knowledge and facilitate discussions about deprescribing or alternative therapies. The framework assumes that demographic variables (such as age and education), clinical conditions (such as polypharmacy or comorbidities), and pharmacist involvement interact to influence both fall risk and older adults’ willingness to take preventive action. A conceptual model was developed to illustrate the hypothesized relationships among demographic and clinical factors, pharmacist counseling, FRID knowledge, and fall risk (Figure 1).

Based on the literature and our conceptual framework, we hypothesized that knowledge of fall-risk-increasing drugs (FRIDs) would be suboptimal among community-dwelling older adults in Jazan. Also, demographic and clinical factors such as age, comorbidities, and polypharmacy would be significantly associated with increased fall risk. Additionally, pharmacist counseling would be positively associated with better FRID knowledge and a greater willingness to discuss medication changes aimed at reducing fall-related harm.

To our knowledge, this is among the first studies in Saudi Arabia to concurrently assess fall risk, knowledge of both prescription and over-the-counter fall-risk-increasing drugs (FRIDs), and the role of pharmacist counseling among community-dwelling older adults. By focusing on a region with unique healthcare delivery challenges and growing aging demographics, this study addresses critical evidence gaps around medication-related fall risks and health literacy. The inclusion of OTC medications and patient-reported willingness to discuss medication changes with pharmacists adds an applied dimension with practical implications for pharmacy-led fall prevention strategies. The findings are expected to inform clinical practice, guide the design of targeted pharmacist interventions, and support national efforts to reduce medication-related harms in older adult populations.

## 2. Material and Methods

### 2.1. Study Design

This cross-sectional survey employed convenience sampling among community-dwelling individuals aged ≥ 60 years in Jazan, a southwestern province of Saudi Arabia. This study was conducted between December 2023 and March 2024.

### 2.2. Measures

The questionnaire was structured in five parts (Supplementary File S1). The initial segment comprised questions about demographics, pre-existing chronic conditions, the number of current prescription and over-the-counter (OTC) drugs, and any pharmacist counseling on medication-related fall risks within the previous six-month period. The second segment included the Stay Independent screening tool designed by the Centers for Disease Control and Prevention, United States of America (USA) under the Stopping Elderly Accidents, Death and Injury (STEADI) initiative which included 12 questions to assess the risk of falling with a total possible score of 14. The first two questions had a score of 2, and the remaining ten questions each had a score of 1. A score of four or more indicates fall risk [26]. The Stay Independent screening tool was not available in Arabic. It was translated by native Arabic-speaking researchers using a forward–backward approach. The translated tool was reviewed by a geriatrician and a pharmacist for clarity and cultural appropriateness. It was then tested with 10 older adults (excluded from the analysis) to ensure understanding and contextual relevance.

Sections three and four of the questionnaire consisted of 15 items assessing knowledge of FRIDs. We used an adapted version of the Falls Risk Awareness Questionnaire from a previous US study for knowledge assessment [14]. Knowledge of P-FRIDs consisted of nine items, whereas knowledge assessment of OTC-FRIDs consisted of six items, with both sections requiring a response of yes, no, or I do not know. Knowledge level was categorized using the Bloom cutoff point, with scores above 80% correct answers indicating good knowledge, 60–80% signifying average knowledge, and below 60% denoting poor knowledge. These thresholds were derived from Bloom’s Taxonomy of Educational Objectives, a widely applied framework in educational and health-related knowledge assessments [27]. The final section contained a single 4-item Likert scale question that asked the respondents about their willingness to discuss medication changes with their pharmacist.

Three experts, comprising one physician, one clinical pharmacist, and a nurse from Jazan University Hospital, Saudi Arabia, assessed the English language of the questionnaire for content and face validity. It was then administered to a professional expert for translation into Arabic followed by back translation to verify and ensure accuracy and consistency across languages.

### 2.3. Survey Recruitment and Administration

The responses were collected through interviews with each respondent. PharmD internship students at the College of Pharmacy, Jazan University, Saudi Arabia, were invited to volunteer for data collection and were informed that they would receive a certificate of appreciation, but no financial incentives. Ten interns were selected on the basis of their communication skills.

Data collectors underwent a 3-day training program. Day 1 included study objectives and ethical considerations. Day 2 covered item-wise questionnaire review, mock interviews, and translation consistency. Day 3 involved role-plays and pilot data collection. Competence was assessed through mock evaluations and feedback sessions. Data collection was conducted in both the urban and rural areas of Jazan. Recruitment occurred during evenings and weekends to maximize coverage. Participants were approached in public places, such as shopping malls, parks, neighborhoods, and places of worship, by trained PharmD internship students who explained the study purpose, assessed eligibility, and obtained verbal consent.

Cognitive ability was subjectively assessed by data collectors using indicators such as coherence in conversation, orientation to place/time, and ability to comprehend questions. Participants who appeared confused or unable to communicate clearly were excluded. This method not only helped to establish rapport with participants, but also identified cognitive limitations to ensure that participants demonstrated the requisite comprehension to understand the questions and complete the questionnaire. Data collectors used an online version of the questionnaire on QualtricsXM (Qualtrics, Provo, UT, USA) to record their responses, which took approximately 15 min to complete. Educational materials from the STEADI initiative were translated into Arabic, printed, and given to the respondents after the interview [28]. Designated study authors were responsible for supervising data collection, reviewing completed forms daily, cross-checking entries, and providing ongoing feedback to data collectors. Discrepancies or ambiguities were immediately clarified with the data collection team.

### 2.4. Ethical Considerations

The study protocol and questionnaire were reviewed and approved by the Standing Committee for Scientific Research at Jazan University, Saudi Arabia (REC-45/03/768). Verbal informed consent was obtained from all participants after explaining the study objectives, confidentiality measures, and voluntary nature of participation. The estimated time for completion of the questionnaire (15 min) was also recorded. The study participants were notified that they could discontinue their participation in the research at any time. Data confidentiality and anonymity were also maintained. Data collectors proceeded with the questionnaire section only for the participants who provided informed consent. All collected data were anonymized. No names or identifiable information were recorded. Unique codes were used for data entry and analysis. Data were stored in encrypted files accessible only to the research team. Physical forms were securely stored and later destroyed after digitization.

### 2.5. Statistical Analysis

Based on Jazan’s population of older adults, a sample size of 384 with a 5% margin of error and 95% confidence interval was estimated using Raosoft online sample size calculator (Raosoft, Inc., Seattle, WA, USA; http://www.raosoft.com/samplesize.html) [29,30]. A 5% margin of error and a 95% confidence interval were used, as these thresholds are commonly employed in public health research to balance precision with feasibility [31]. A total of 490 individuals were approached, of which 432 consented to participate. Based on subjective evaluation by data collectors, 41 participants were excluded (30 declined to participate and 11 withdrew before completing the questionnaire), whereas 391 participants were included in the study (79.8% response rate). Responses of participants who withdrew from the study before completion were later deleted and were not part of the data analyses. Minimal missing data were encountered due to supervised, in-person data collection. When missing responses occurred, list wise deletion was applied. Three study authors were assigned to ensure data integrity through double-checking and data cleaning before analysis. Descriptive statistics depicted sample characteristics reported as frequencies, percentages, means, and standard deviations. To assess the adjusted associations between the explanatory variables and fall risk, multivariable logistic regression analysis was performed. Multicollinearity was assessed using variance inflation factors (VIFs). To assess the probability of categorization into various groups related to knowledge of P-FRIDs and OTC-FRIDs as well as the willingness to change medications, we employed an ordered probit regression analysis [32]. To verify the model’s compliance with the proportional odds/parallel lines assumption, we employed the gologit2 function in STATA 18 and conducted a Wald test on the final model. This approach allowed us to confirm that the assumption was met [33]. Calculations were performed to determine the marginal impacts of each independent variable on the ordered probabilities of knowledge and willingness. For these ordered probit models, for each chronic condition, the reference was not having that particular chronic condition. All statistical analyses were performed using STATA version 18. Results were considered significant at *p* ≤ 0.05. No separate power calculations were conducted for subgroup analyses; these were exploratory and based on available sample sizes.

## 3. Results

### 3.1. Sample Characteristics

**Figure 2:**
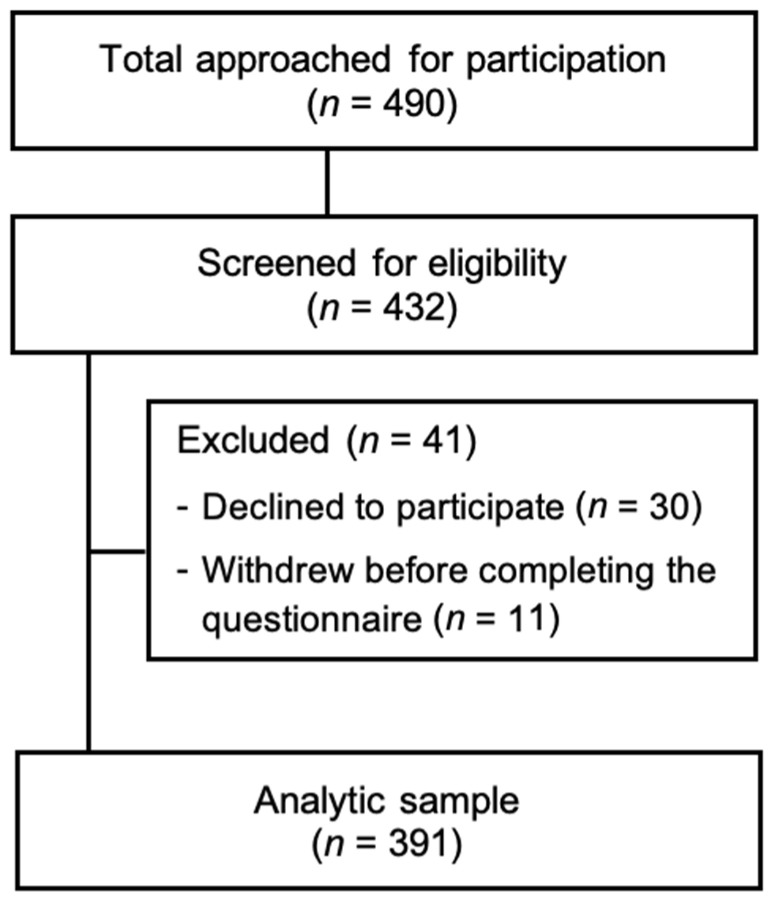
Participant recruitment.

Figure 2 provides a CONSORT-style flow diagram detailing the recruitment process and the final sample. The mean (SD) age of participants was 68.9 (6.0) years. The majority of the respondents (32%) were aged 66–70 years, 57.8% were male, 88.2% were married, and 66.2% were using one to four prescription medications. Less than half (43%) were not using any OTC medications, while 58.8% had received counseling from pharmacists regarding fall risk medications within the previous six months. Hypertension (56.5%), diabetes (49.9%), and vision problems (39.4%) were the most common chronic conditions observed. Table 1 lists detailed demographic characteristics.

### 3.2. Risk of Fall

The Stay Independent Scale score indicated that approximately 57% of the respondents scored 4 or higher and were determined to be at a risk of falling. More than one-third (38.6%) reported falling in the past year, and 66.8% were concerned about it (Supplementary Table S1). Table 2 presents the association between the explanatory variables and fall risk using multivariable logistic regression. Age was associated with a higher risk of falls (95% CI, 1.00–1.14; *p* = 0.05). Arthritis (95% CI, 2.51–13.06; *p* < 0.001) and obesity (95% CI, 2.33–15.46; *p* < 0.001) were significantly associated with an increased fall risk, as was diabetes (95% CI, 1.38–5.64; *p* = 0.004). No other comorbidities in our sample, except the “other” category (including cancer, stroke effects, and epilepsy), showed a significant fall risk (95% CI, 3.31–305.12; *p* = 0.003). Notably, those receiving pharmacist counseling also had a substantial risk of falling (95% CI, 3.45–13.51; *p* < 0.001).

We examined the relationship between the number of medications taken and fall risk score. Supplementary Figure S1 illustrates the relationship between medication burden and fall risk score using box plots. Fall risk scores were positively associated with the number of medications taken, with higher median scores observed among those using ≥5 prescription or OTC medications. Supplementary Figure S2 displays a forest plot of adjusted odds ratios for significant fall risk predictors. Higher education, pharmacist counseling, hearing loss, bladder or bowel incontinence, and specific chronic conditions such as arthritis, diabetes and obesity emerged as strong contributors.

### 3.3. Knowledge Assessment of P-FRIDs

Supplementary Table S2 summarizes the assessment results for knowledge of P-FRIDs. An item-level analysis of P-FRID knowledge responses revealed that drugs used for bladder symptoms (anticholinergics) and antipsychotics (sedatives) were the least recognized P-FRIDs. Based on Bloom’s cutoff point, only 11.5% of the respondents had good knowledge of P-FRIDs. Approximately 47.1% had average knowledge, and 41.4% had poor knowledge. Table 3 shows the estimated marginal effects of the explanatory variables on the probabilities of various P-FRID knowledge levels, as determined by the ordered probit models. Individuals without chronic conditions were 6.6% less likely (95% CI, −0.128–−0.004; *p* = 0.04) to possess good knowledge of P-FRIDs. Additionally, individuals who had five or more prescription medications were 20% more likely (95% CI, 0.001–0.405; *p* = 0.05) to have poor knowledge than those who had no prescription medications. However, individuals who received pharmacist counseling were 12.4% more likely (95% CI, 0.079–0.169, *p* < 0.001) to have better knowledge than those who did not.

### 3.4. Knowledge Assessment of OTC-FRIDs

Supplementary Table S3 presents the knowledge assessment of the OTC-FRIDs. Among OTC medications, sedating antihistamines and sleep aids/cold medications were most commonly missed. High knowledge levels were demonstrated by 24.6% of respondents, whereas 23.8% exhibited poor knowledge. Table 4 presents the estimated marginal effects of the ordered probit models on the probabilities of the various OTC-FRID knowledge levels. Individuals with primary school education were 9.1% more likely (95% CI, 0.004–0.179; *p* = 0.04) to possess good knowledge of OTC-FRIDs. Those with household incomes of Saudi Arabian Riyals (SAR) 10,000–15,000 and over SAR 15,000 were 16.2% (95% CI, 0.067–0.257; *p* < 0.001) and 12.8% (95% CI, 0.009–0.246; *p* = 0.03) more likely, respectively, to have good knowledge (a currency exchange rate of SAR 1 = USD 0.27 is applicable). Conversely, obese individuals were 8.3% less likely (95% CI, −0.158–−0.008; *p* = 0.03) to have good knowledge of OTC-FRIDs. No association was found between pharmacist counseling and knowledge related to OTC-FRIDs.

Supplementary Figure S3 comparing FRID knowledge across demographic groups revealed that “average” knowledge was the most frequently reported level across all strata. Overall, participants demonstrated limited FRID knowledge pertaining to CNS-affecting drugs such as antipsychotics and sedating antihistamines (Supplementary Tables S2 and S3). This highlights that participants were more knowledgeable about medications used for chronic physical conditions but demonstrated limited awareness of FRIDs related to CNS effects. Recognition of OTC-FRIDs, particularly sedating antihistamines and sleep aids, was noticeably lower, suggesting the need for greater public awareness regarding the fall risks associated with commonly used non-prescription drugs. These findings emphasize the need for targeted educational interventions focusing on high-risk drug classes that are often overlooked.

### 3.5. Willingness to Discuss Medication Changes with a Pharmacist

Respondents’ willingness to discuss changes in their medication or dose reduction with regard to FRIDs was also explored. Nearly 75% of the respondents were open to discussing medication changes with pharmacists to reduce fall risk. Ordered probit models were used to calculate the estimated marginal effects of the explanatory variables on the probability of respondents’ willingness to discuss medication changes with a pharmacist; the results are presented in Table 5. Individuals who were single were 11.4% less likely (95% CI, −0.224–−0.004; *p* = 0.04) whereas those with a bachelor’s degree or higher were 18.2% more likely (95% CI, 0.037–0.326; *p* = 0.01) to be in the very likely category to discuss medication changes with a pharmacist. Individuals with vision problems were 12.4% more likely (95% CI, 0.042–0.207; *p* = 0.003) to be in the very likely category. Moreover, individuals who received pharmacist counseling were 9.4% more likely (95% CI, 0.020–0.167; *p* = 0.01) to be in the very likely category than those who did not receive pharmacist counseling.

## 4. Discussion

### 4.1. Key Findings

This study assessed participants’ risk of falls, knowledge levels of fall-associated medications, impact of pharmacist counseling, and willingness to discuss medication changes with pharmacists. Overall, 57% of the respondents were at risk of falling, and more than one-third reported falling in the past year. In addition to age, arthritis, obesity, and diabetes increase the likelihood of fall risk. Good knowledge of P-FRIDs and OTC-FRIDs was demonstrated by only a few respondents. Those who received pharmacist counseling were more likely to have good knowledge of P-FRIDs and were willing to discuss medication changes with their pharmacist.

### 4.2. Review of Existing Literature

More than half of the study population (56.5%) were at risk of falling. The prevalence of falls in our study was 39% which exceeded the global prevalence of 26.5% reported in a recent systematic review and meta-analysis [5]. However, among the GCC countries, the pooled prevalence of falls was estimated to be 46.9% [7]. Our study’s findings indicated a lower incidence of falls compared to studies conducted in the Western [34] and Central regions (49.9%) [35], but higher than that in a Northwestern study (25.2%) [36] in Saudi Arabia. Moreover, the fall prevalence in our study was higher than that in studies from the USA (27.6%) [37], the UK (28%) [38], and Egypt (29%) [39]. This observed variation in the prevalence of falls across countries may be influenced by factors such as differences in population demographics, lifestyle, prevalence of chronic conditions, and social support systems. Fear of falling was reported by approximately two-thirds (67%) of the participants, in contrast to findings from Western Saudi Arabia, where less than one-third (31.8%) were reported to live with this fear [34]. The fear of falling may be heightened in those who have experienced a fall previously and can result in a loss of confidence and fear of performing daily activities [6]. More than half of the respondents in our study received counseling from a pharmacist regarding FRIDs and fall-prevention strategies. Pharmacist counseling has been shown to effectively reduce medication-related problems, thereby improving clinical outcomes and decreasing the incidence of falls [40].

Age was significantly associated with an increased risk of falls, which is consistent with the results of a comprehensive review and meta-analysis of thirty-one studies [3]. The association between increasing age and fall risk reached the threshold of statistical significance (OR 1.07; 95% CI, 1.00–1.14; *p* = 0.05), suggesting a modest effect. While this may appear marginal, even small incremental risks per year can have substantial implications at the population level—especially in rapidly aging societies. Thus, the finding remains clinically relevant and supports continued emphasis on age-adapted fall prevention strategies. Additionally, we found a similar risk of falls among individuals with arthritis, obesity, and diabetes. Previous studies have reported an increased risk of falling among older individuals with arthritis and obesity [41,42]. A systematic review and meta-analysis that examined six studies involving 14,685 participants concluded that diabetes is associated with a greater fall risk [43]. Diabetes is a contributing factor to falls due to the potential of diabetes medications to induce hypoglycemia, leading to impaired balance and subsequent falls [44]. Interestingly, those counseled by pharmacists had a significantly higher fall risk. This unexpected association may be due to unmeasured factors, such as differences in chronic conditions, medication regimens, and quality of counseling. Additionally, pharmacists may have counseled individuals who were perceived to be at a higher fall risk.

This study evaluated older adults’ knowledge of P-FRIDs. Common P-FRIDs include hypnotics, antihypertensives, anxiolytics, antidepressants, antidiabetic and anticholinergic drugs [45]. Only 11.5% of the participants exhibited adequate knowledge of P-FRIDs. Comparable findings were observed in a separate study, which revealed that numerous older adults were unaware of the contribution of medication use to falls [46]. Furthermore, our findings indicate that older individuals without pre-existing chronic conditions are less likely to have good knowledge of P-FRIDs, as they would be taking lesser prescription medications. Polypharmacy increases the risk of falls [47]. In our study, older individuals taking five or more medications were 20% more likely to have poor knowledge of P-FRIDs than those who did not take any prescription medication. This finding is of concern, considering the results of studies on polypharmacy and fall risk. For instance, one study reported a 2% increase in injurious falls with each additional prescribed drug [47]. Another study reported a 14.8 percentage point increase in hospital admissions due to falls in those taking ten or more medications compared to 1.5% in those taking no medications [48]. Awareness of P-FRIDs is crucial for mitigating the fall risks associated with polypharmacy. Our findings indicated that participants who received counseling from pharmacists were more likely to possess a good understanding of P-FRIDs, which is consistent with a recent study showing that pharmacist-led educational interventions improve their understanding of fall-related medications [49].

Certain OTC medications may be inappropriate or risky for older adults because of their perceived safety and accessibility, despite the potential risks [50]. In our study, only a quarter of the participants demonstrated good knowledge of OTC-FRIDs, which was superior than their knowledge of P-FRIDs. Substantial evidence regarding knowledge assessment of OTC-FRIDs is limited. Interestingly, participants demonstrated higher knowledge of OTC-FRIDs than P-FRIDs (24.6% vs. 11.5%). Leonetti and Lee (2014) reported that respondents demonstrated better knowledge of OTC-FRIDs compared to P-FRIDs [14]. This may reflect greater attention to OTC product labels and advertisements, which often highlight safety warnings. A recent study showed that while OTC drug use is highly prevalent, particularly analgesics, consumption decreases with age. Among those aged 71 and older, 36.4% reported using OTC drugs. This indicates that older adults may have knowledge regarding these medications [51]. OTC medications are frequently perceived as inherently safer, leading to increased user vigilance, but not necessarily accurate risk assessment [50]. In contrast, prescription medication risks are less visible to patients, especially when consultations are brief and are typically dictated by healthcare providers, with limited opportunity for patient-led review. These behavioral differences may partly explain why OTC-related risks are more readily identified. Nevertheless, good knowledge levels for both categories were low, underscoring the need for unified education strategies encompassing all FRID classes. The present study indicated that individuals with primary school education and higher household income were more likely to possess good knowledge of OTC-FRIDs, suggesting higher health literacy and access to educational resources. Furthermore, receiving pharmacist counseling was not likely to result in better knowledge of OTC-FRIDs, potentially due to the high workload of community pharmacists, resulting in time constraints, as reported in a study on the implementation of a fall prevention service [52].

In our study, three-fourths of the respondents showed willingness to discuss medication change with their pharmacist, similar to another study where most of the individuals were willing to change an FRID upon a healthcare provider’s recommendation [53]. In our study, individuals with a bachelor’s degree or higher showed a greater likelihood of discussing medication changes with their pharmacist. Higher educational level was associated with individuals’ willingness to use pharmacy services [54]. In addition, those who received pharmacist counseling were more likely to be in the very likely category to discuss medication changes with their pharmacists, as pharmacist-led interventions regarding advice on medication changes have been shown to potentially lower the risk of falls [55].

Our findings align with those of an Indian study that reported lower awareness among older adults regarding medications and polypharmacy [56]. Contrasting results were reported by another study, in which respondents demonstrated higher knowledge scores for medications [57]. However, the magnitude of unawareness observed in our sample may be attributable to systemic factors, including limited pharmacist-led education in community settings and insufficient integration of geriatric pharmaceutical services into primary care.

Cultural norms in Saudi Arabia—such as reliance on family caregivers, gender segregation in healthcare settings, and the use of traditional medicine—may influence health literacy, risk perception, and healthcare-seeking behavior. A study conducted in Saudi Arabia found that the majority of caregivers were family members. Moreover, these caregivers expressed a need for regular healthcare services for themselves and a home health visit service to assist them in caring for their older relatives [58]. The majority of older adults, including family caregivers, are not aware of the risk of falling and do not consider falls to be a significant concern [59]. A recent study reported that half of the respondents did have a specific gender preference for a physician; however, personal care and invasive procedures had a pronounced gender preference [60]. In Saudi Arabia, the use of herbal medicines is widespread among the population, which is deeply rooted in cultural and religious practices. In rural regions, these remedies are often the preferred option when individuals fall ill, and they typically do not seek medical advice before using them [61]. Indiscriminate use of herbal medications may add unwanted fall risk to older adults. Religious practices such as prayer movements may heighten fall risk for frail individuals as they are less capable of maintaining stability during movements or managing sudden shifts in posture, which are sometimes required during prayer rituals [62]. However, this may be rarely addressed in counseling.

### 4.3. Interesting Findings and Their Interpretation

An interesting finding was that pharmacist counseling was associated with higher reported fall risk. One plausible explanation is reverse causality—individuals already at risk of falls may be more likely to receive counseling as a targeted intervention. Given the cross-sectional design, we cannot ascertain the directionality of this association. Confounding factors such as polypharmacy, multi-morbidity, and severity of illness may also contribute, as pharmacists may selectively counsel more vulnerable patients.

Moreover, variability in the content, duration, and delivery of counseling across settings could dilute its potential impact [63]. This may also be due to community pharmacists’ high workload and staff shortages, leading to a lack of time for effective counseling [52]. Inconsistent messaging or lack of reinforcement from multidisciplinary teams may limit effectiveness. Finally, counseling may inadvertently result in risk compensation behaviors, wherein patients, feeling reassured, may engage in activities that elevate fall risk. A scoping review on adult inpatients’ perceptions of fall risk found that many patients did not accurately perceive their fall risk compared to healthcare professionals’ assessments. This indicates that while patients may overestimate safety after fall prevention counseling, they might not modify their behavior to reduce fall risk [64]. These nuances warrant further longitudinal and qualitative investigations.

### 4.4. Strengths and Limitations

This study has several strengths, including the use of a structured tool to assess fall risk and medication knowledge, a diverse sample of older adults from both urban and rural settings in the Jazan region, and a focus on both prescription and OTC medication risks. However, there are also important limitations. The use of convenience sampling may have introduced selection bias, limiting the representativeness of the findings. The sample may over-represent individuals who are more willing to engage with healthcare services, and the absence of randomization restricts the generalizability of the results. Additionally, post-stratification weighting was not performed; hence, demographic representativeness may be influenced by the inherent biases of convenience sampling. Sensitivity analyses using alternative sets of variables were not conducted in this study. However, future analyses could benefit from such approaches to test the robustness of findings across different modeling strategies.

Moreover, data were based on self-reported measures, making them subject to recall bias—particularly for falls reported over the past year and medication use patterns. Another limitation is the absence of a formal cognitive screening tool during participant recruitment. Although interviewers excluded individuals with apparent cognitive impairment, this approach relied on subjective judgment rather than validated assessment scales. This may have led to the inclusion of participants with undiagnosed mild cognitive impairment, potentially affecting the accuracy of self-reported data regarding medication use and fall history. Future studies should consider incorporating brief, validated cognitive screening instruments to enhance data reliability. Additionally, while we assessed whether pharmacist counseling was received, we were unable to evaluate the quality, duration, or content of these sessions, which are critical variables in determining their effectiveness. Formal validation metrics for the Arabic version of the Stay Independent screening tool are currently unavailable. This represents a limitation, as reliability coefficients, sensitivity, and specificity have not been established in Arabic-speaking populations. Finally, as a cross-sectional study conducted in a single region, the temporal direction of associations cannot be established, and the findings may not be generalizable to the broader Saudi older adult population. Despite these limitations, this study provides novel insights into medication-related fall risk in an under-researched setting and highlights critical areas for clinical and policy actions.

## 5. Conclusions

This study demonstrates that knowledge of fall-risk-increasing drugs (FRIDs), particularly prescription medications, remains limited among older adults in the Jazan region. Furthermore, willingness to change medications and receiving pharmacist counseling were significantly associated with an individual’s fall risk status. Our findings reveal that older adults who received pharmacist counseling were more likely to report a higher fall risk, suggesting that pharmacist involvement currently occurs in response to, rather than as a preventive measure against, fall risks. While higher knowledge of OTC-FRIDs was observed compared to P-FRIDs, both remain suboptimal, especially among individuals with chronic conditions such as diabetes and arthritis.

Based on these insights, we propose the implementation of targeted medication reviews focusing on OTC medications in patients with arthritis and diabetes. It is also recommended to develop standardized pharmacist counseling protocols that specifically address fall risks. Furthermore, incorporating the Stay Independent screening tool into routine pharmaceutical care for older adults would facilitate a straightforward and comprehensive assessment of fall risk. Future research should focus on longitudinal studies to assess the temporal relationship between pharmacist counseling and fall outcomes. In addition, intervention studies comparing different educational approaches for improving FRID knowledge would be beneficial. Qualitative research exploring barriers to medication safety discussions between patients and pharmacists also would provide deeper insights to understand these complex behaviors. These directions are essential to validate our findings and build comprehensive, patient-centered fall prevention strategies.

In line with our conceptual framework, this study confirms the interconnected role of demographic, clinical, and behavioral factors—particularly pharmacist engagement—in shaping fall risk outcomes. By addressing knowledge gaps and fostering interdisciplinary fall prevention models, our study contributes novel evidence to the growing literature on medication safety among older adults in the Middle East.

## Figures and Tables

**Table 1:** Baseline characteristics.

Characteristic	No. (%) (N = 391) ^a^
Gender	
Male	226 (57.8)
Female	165 (42.2)
Marital status	
Married	344 (88.2)
Single	27 (6.9)
Divorced/Widow	19 (4.9)
Educational status	
None	138 (35.4)
Primary School	103 (26.4)
Secondary School	96 (24.6)
Bachelor and above	53 (13.6)
Employment status	
Not working	351 (89.8)
Still working	40 (10.2)
Household income (SAR)	
<5000	92 (23.5)
5000–9999	113 (28.9)
10,000–15,000	130 (33.2)
>15,000	56 (14.3)
Existing chronic conditions	
Vision problems	154 (39.4)
Hearing loss	35 (9.0)
Back problems	105 (26.9)
Arthritis	120 (30.7)
Hypertension	221 (56.5)
Heart disease	70 (17.9)
Osteoporosis	52 (13.3)
Bladder or bowel incontinence	21 (5.4)
Diabetes	195 (49.9)
Obesity	79 (20.2)
None	34 (8.7)
Other	24 (6.1)
Prescription medications	
0	57 (14.6)
1–4	260 (66.5)
≥5	74 (18.9)
OTC medications	
0	168 (42.5)
1–2	96 (24.3)
3–4	83 (21.0)
≥5	48 (12.2)
Received pharmacist counseling	
No	161 (41.2)
Yes	230 (58.8)
Fall Risk	
No risk	170 (43.5)
At risk	221 (56.5)
Knowledge of P-FRIDs	
Poor	162 (41.4)
Average	184 (47.1)
Good	45 (11.5)
Knowledge of OTC-FRIDs	
Poor	93 (23.8)
Average	202 (51.7)
Good	96 (24.6)
Willingness to discuss medication changes with pharmacist	
Very unlikely	27 (6.9)
Somewhat unlikely	72 (18.4)
Somewhat likely	188 (48.1)
Very likely	104 (26.6)

Abbreviations: SAR: Saudi Arabian Riyals; OTC: over-the-counter; P-FRIDs: prescription fall-risk-increasing drugs; OTC-FRIDs: over-the-counter fall-risk-increasing drugs. ^a^ Percentages were based on the number of responses to each question.

**Table 2:** Association between the explanatory variables and fall risk using bivariate regression (N = 391).

Explanatory Variables	OR (95% CI)	SE	z-Score	*p* Value
Age	1.07 (1.00–1.14)	0.03	1.97	0.05 *
Gender				
Male	Reference			
Female	1.13 (0.57–2.22)	0.39	0.35	0.73
Marital status				
Married	Reference			
Single	0.73 (0.19–2.28)	0.51	−0.45	0.65
Divorced/Widow	0.35 (0.09–1.36)	0.24	−1.52	0.13
Educational status				
None	Reference			
Primary School	0.68 (0.30–1.54)	0.28	−0.93	0.35
Secondary School	0.67 (0.29–1.55)	0.29	−0.93	0.35
Bachelor and above	2.77 (0.78–9.85)	1.79	1.57	0.12
Employment status				
Not working	Reference			
Still working	0.33 (0.10–1.06)	0.20	−1.86	0.06
Household income (SAR)				
<5000	Reference			
5000–9999	0.76 (0.31–1.83)	0.34	−0.62	0.54
10,000–15,000	0.73 (0.28–1.86)	0.35	−0.66	0.51
>15,000	0.56 (0.18–1.78)	0.33	−0.99	0.32
Existing chronic conditions ^a^				
Vision problems	1.04 (0.52–2.09)	0.37	0.11	0.91
Hearing loss	3.23 (0.86–12.04)	2.17	1.74	0.08
Back problems	1.26 (0.55–2.85)	0.52	0.55	0.56
Arthritis	5.73 (2.51–13.06)	2.41	4.15	<0.001 ***
Hypertension	1.50 (0.75–3.01)	0.53	1.15	0.25
Heart disease	1.44 (0.57–3.66)	0.69	0.77	0.44
Osteoporosis	1.35 (0.47–3.92)	0.73	0.56	0.58
Bladder or bowel incontinence	3.58 (0.60–21.51)	3.28	1.40	0.16
Diabetes	2.79 (1.38–5.64)	1.00	2.86	0.004 **
Obesity	6.00 (2.33–15.46)	2.90	3.71	<0.001 ***
None	1.27 (0.32–5.07)	0.90	0.34	0.74
Other	31.76 (3.31–305.12)	36.66	3.00	0.003 **
Prescription medications				
0	Reference			
1–4	1.54 (0.51–4.70)	0.88	0.76	0.45
≥5	0.88 (0.19–4.03)	0.68	−0.17	0.87
OTC medications				
0	Reference			
1–2	1.38 (0.65–2.96)	0.54	0.84	0.40
2–4	1.83 (0.80–4.20)	0.77	1.42	0.16
≥5	2.21 (0.73–6.68)	1.25	1.41	0.16
Knowledge of P-FRIDs				
Poor	Reference			
Average	1.88 (0.93–3.79)	0.67	1.77	0.08
Good	0.55 (0.19–1.62)	0.30	−1.08	0.28
Knowledge of OTC-FRIDs				
Poor	Reference			
Average	0.83 (0.37–1.85)	0.34	−0.46	0.65
Good	0.27 (0.10–0.70)	0.13	−2.68	0.007 *
Received pharmacist counseling				
No	Reference			
Yes	6.83 (3.45–13.51)	2.38	5.51	<0.001 ***

Abbreviations: OR: odds ratio; CI: confidence interval; SE: standard error; SAR: Saudi Arabian Riyals; OTC: over-the-counter; P-FRIDs: prescription fall-risk-increasing drugs; OTC-FRIDs: over-the-counter fall-risk-increasing drugs. ^a^ For this variable, the reference was “not having that particular chronic condition”. *** *p* < 0.001; ** 0.001 ≤ *p* < 0.01; * 0.01 ≤ *p* < 0.05.

**Table 3:** Estimated marginal effect (EME) of the explanatory variables on knowledge of P-FRIDs probabilities (N = 391).

Explanatory Variables	Knowledge of P-FRIDs [EME (95% CI)]		
	Poor Knowledge	Average Knowledge	Good Knowledge
Age	−0.005 (−0.013–0.003)	0.002 (−0.002–0.006)	0.002 (−0.002–0.006)
Gender			
Male	Reference		
Female	0.072 (−0.017–0.162)	−0.036 (−0.082–0.010)	−0.036 (−0.081–0.009)
Marital status			
Married	Reference		
Single	−0.106 (−0.263–0.051)	0.043 (−0.006–0.092)	0.062 (−0.048–0.172)
Divorced/Widow	−0.087 (−0.278–0.104)	0.037 (−0.029–0.104)	0.049 (−0.076–0.175)
Educational status			
None	Reference		
Primary School	0.033 (−0.078–0.144)	−0.018 (−0.078–0.042)	−0.015 (−0.066–0.036)
Secondary School	−0.037 (−0.151–0.077)	0.017 (−0.036–0.071)	0.019 (−0.041–0.080)
Bachelor and above	−0.058 (−0.215–0.099)	0.026 (−0.040–0.093)	0.032 (−0.060–0.123)
Employment status			
Not working	Reference		
Still working	0.125 (−0.037–0.287)	−0.072 (−0.179–0.034)	−0.052 (−0.109–0.005)
Household income (SAR)			
<5000	Reference		
5000–9999	0.104 (−0.010–0.218)	−0.048 (−0.101–0.005)	−0.056 (−0.120–0.008)
10,000–15,000	0.080 (−0.044–0.203)	−0.035 (−0.089–0.019)	−0.045 (−0.116–0.026)
>15,000	0.101 (−0.052–0.255)	−0.047 (−0.122–0.029)	−0.055 (−0.135–0.026)
Existing chronic conditions ^a^			
Vision problems	−0.071 (−0.166–0.023)	0.035 (−0.011–0.080)	0.037 (−0.013–0.087)
Hearing loss	−0.071 (−0.217–0.076)	0.031 (−0.025–0.087)	0.040 (−0.052–0.131)
Back problems	0.054 (−0.055–0.164)	−0.028 (−0.086–0.031)	−0.026 (−0.078–0.031)
Arthritis	0.023 (−0.085–0.130)	−0.011 (−0.066–0.043)	−0.011 (−0.064–0.041)
Hypertension	−0.036 (−0.134–0.062)	0.018 (−0.032–0.068)	0.018 (−0.031–0.066)
Heart disease	−0.038 (−0.157–0.080)	0.018 (−0.035–0.071)	0.020 (−0.045–0.086)
Osteoporosis	0.040 (−0.094–0.0174)	−0.021 (−0.094–0.053)	−0.019 (−0.080–0.042)
Bladder or bowel incontinence	0.068 (−0.124–0.260)	−0.037 (−0.151–0.077)	−0.031 (−0.110–0.048)
Diabetes	0.035 (−0.059–0.128)	−0.017 (−0.064–0.029)	−0.018 (−0.065–0.030)
Obesity	0.009 (−0.097–0.0115)	−0.004 (−0.058–0.049)	−0.004 (−0.057–0.048)
None	0.168 (−0.032–0.369)	−0.102 (−0.243–0.039)	−0.066 * (−0.128–−0.004)
Other	0.058 (−0.124–0.240)	−0.031 (−0.136–0.074)	−0.027 (−0.104–0.050)
Prescription medications			
0	Reference		
1–4	0.046 (−0.108–0.200)	−0.018 (−0.071–0.035)	−0.028 (−0.130–0.073)
≥5	0.203 * (0.001–0.405)	−0.108 * (−0.208–−0.008)	−0.096 (−0.207–0.016)
OTC medications			
0	Reference		
1–2	0.016 (−0.092–0.123)	−0.008 (−0.066–0.049)	−0.007 (−0.066–0.049)
3–4	−0.052 (−0.163–0.060)	0.024 (−0.027–0.076)	0.027 (−0.034–0.088)
≥5	−0.047 (−0.188–0.094)	0.022 (−0.042–0.086)	0.025 (−0.053–0.102)
Received pharmacist counseling			
No	Reference		
Yes	−0.289 *** (−0.385–−0.194)	0.165 *** (0.100–0.231)	0.124 *** (0.079–0.169)

Abbreviations: SAR: Saudi Arabian Riyals; OTC: over-the-counter; P-FRIDs: prescription fall-risk-increasing drugs; OTC-FRIDs: over-the-counter fall-risk-increasing drugs. ^a^ For this variable, the reference was “not having that particular chronic condition”. Asterisks represent significant group differences compared to the reference based on the ordered probit regression model and the outcome (poor knowledge, average knowledge, good knowledge): *** *p* < 0.001; * 0.01 ≤ *p* < 0.05.

**Table 4:** Estimated marginal effect (EME) of the explanatory variables on knowledge of OTC-FRIDs probabilities (N = 391).

Explanatory Variables	Knowledge of OTC-FRIDs [EME (95% CI)]		
	Poor Knowledge	Average Knowledge	Good Knowledge
Age	−0.005 (−0.011–0.001)	<0.000 (−0.001–0.001)	0.005 (−0.001–0.011)
Gender			
Male	Reference		
Female	−0.045 (−0.115–0.024)	−0.002 (−0.009–0.006)	0.047 (−0.026–0.120)
Marital status			
Married	Reference		
Single	0.035 (−0.106–0.177)	−0.001 (−0.016–0.013)	−0.034 (−0.162–0.094)
Divorced/Widow	0.081 (−0.103–0.265)	−0.010 (−0.056–0.037)	−0.071 (−0.210–0.068)
Educational status			
None	Reference		
Primary School	−0.089 * (−0.173–−0.005)	−0.002 (−0.020–0.016)	0.091 * (0.004–0.179)
Secondary School	−0.068 (−0.160–0.024)	0.002 (−0.011–0.015)	0.066 (−0.024–0.157)
Bachelor and above	−0.033 (−0.165–0.099)	0.003 (−0.009–0.016)	0.029 (−0.092–0.151)
Employment status			
Not working	Reference		
Still working	0.110 (−0.026–0.246)	−0.016 (−0.057–0.025)	−0.094 (−0.191–0.003)
Household income (SAR)			
<5000	Reference		
5000–9999	−0.098 (−0.203–0.007)	0.022 (−0.011–0.055)	0.076 (−0.004–0.155)
10,000–15,000	−0.172 ** (−0.279–−0.066)	0.010 (−0.027–0.046)	0.162 *** (0.067–0.257)
>15,000	−0.146 * (−0272–−0.019)	0.018 (−0.018–0.054)	0.128 * (0.009–0.246)
Existing chronic conditions ^a^			
Vision problems	0.037 (−0.0038–0.112)	<0.000 (−0.005–0.006)	−0.037 (−0.111–0.036)
Hearing loss	−0.057 (−0.162–0.049)	−0.008 (−0.038–0.022)	0.065 (−0.070–0.199)
Back problems	0.029 (−0.058–0.117)	<0.000 (−0.006–0.005)	−0.029 (−0.113–0.054)
Arthritis	0.070 (−0.019–0.159)	−0.003 (−0.017–0.010)	−0.067 (−0.147–0.013)
Hypertension	−0.023 (−0.099–0.053)	<0.000 (−0.004–0.003)	0.023 (−0.053–0.100)
Heart disease	−0.033 (−0.121–0.054)	−0.002 (−0.015–0.010)	0.036 (−0.064–0.135)
Osteoporosis	0.021 (−0.085–0.128)	<0.000 (−0.006–0.006)	−0.021 (−0.122–0.081)
Bladder or bowel incontinence	−0.017 (−0.162–0.128)	−0.001 (−0.015–0.013)	0.018 (−0.140–0.176)
Diabetes	−0.005 (−0.078–0.069)	<0.000 (−0.002–0.002)	0.005 (−0.070–0.079)
Obesity	0.092 (−0.001–0.184)	−0.009 (−0.031–0.013)	−0.083 * (−0.158–−0.008)
None	0.023 (−0.130–0.176)	−0.001 (−0.010–0.009)	−0.023 (−0.166–0.121)
Other	0.137 (−0.032–0.307)	−0.028 (−0.094–0.039)	−0.110 * (−0.215–−0.004)
Prescription medications			
0	Reference		
1–4	−0.072 (−0.208–0.064)	−0.001 (−0.020–0.018)	0.073 (−0.050–0.196)
≥5	0.068 (−0.122–0.258)	−0.018 (−0.067–0.032)	−0.050 (−0.194–0.094)
OTC medications			
0	Reference		
1–2	0.029 (−0.054–0.112)	0.001 (−0.004–0.006)	−0.030 (−0.114–0.054)
3–4	0.051 (−0.040–0.142)	−0.001 (−0.011–0.009)	−0.050 (−0.136–0.036)
≥5	−0.009 (−0.114–0.096)	−0.001 (−0.016–0.014)	0.010 (−0.110–0.131)
Received pharmacist counseling			
No	Reference		
Yes	−0.015 (−0.086–0.056)	<0.000 (−0.002–0.002)	0.015 (−0.057–0.087)

Abbreviations: SAR: Saudi Arabian Riyals; OTC: over-the-counter; P-FRIDs: prescription fall-risk-increasing drugs; OTC-FRIDs: over-the-counter fall-risk-increasing drugs. ^a^ For each chronic condition, the reference was “not having that particular chronic condition”. Asterisks represent significant group differences compared to the reference based on the ordered probit regression model and the outcome (poor knowledge, average knowledge, good knowledge): *** *p* < 0.001; ** 0.001 ≤ *p* < 0.01; * 0.01 ≤ *p* < 0.05.

**Table 5:** Estimated marginal effect (EME) of the explanatory variables on willingness to discuss medication changes with the pharmacist probabilities (N = 391).

Explanatory Variables	Willingness to Discuss Medication Changes with a Pharmacist [EME (95% CI)]			
	Very Unlikely	Somewhat Unlikely	Somewhat Likely	Very Likely
Age	−0.001 (−0.003–0.002)	−0.001 (−0.005 0.003)	<0.000 (−0.000–0.000)	0.001 (−0.005–0.008)
Gender				
Male	Reference			
Female	0.002 (−0.028–0.032)	0.003 (−0.040–0.046)	<0.000 (−0.001–0.001)	−0.005 (−0.079–0.069)
Marital status				
Married	Reference			
Single	0.062 (−0.023–0.147)	0.073 (−0.005–0.151)	−0.021 (−0.075–0.033)	−0.114 * (−0.224–−0.004)
Divorced/Widow	−0.064 (−0.039–0.167)	0.075 (−0.016–0.166)	−0.023 (−0.089–0.044)	−0.117 (−0.045–0.012)
Educational status				
None	Reference			
Primary School	−0.028 (−0.069–0.013)	−0.035 (−0.086–0.016)	0.005 (−0.007–0.018)	0.057 (−0.027–0.142)
Secondary School	−0.036 (−0.078–0.006)	−0.047 (−0.101–0.007)	0.004 (−0.010–0.018)	0.079 (−0.013–0.171)
Bachelor and above	−0.063 ** (−0.109–−0.017)	−0.097 ** (−0.167–−0.027)	−0.021 (−0.069–0.026)	0.182 * (0.037–0.326)
Employment status				
Not working	Reference			
Still working	0.054 (−0.018–0.127)	0.066 (−0.005–0.137)	−0.017 (−0.060–0.025)	−0.103 * (−0.204–−0.002)
Household income (SAR)				
<5000	Reference			
5000–9999	−0.003 (−0.037–0.031)	−0.005 (−0.061–0.050)	−0.001 (−0.011–0.009)	0.009 (−0.090–0.109)
10,000–15,000	0.014 (−0.027–0.054)	0.020 (−0.040–0.081)	0.001 (−0.006–0.008)	−0.035 (−0.140–0.070)
>15,000	0.031 (−0.026–0.087)	0.042 (−0.032–0.115)	−0.003 (−0.021–0.014)	−0.069 (−0.189–0.051)
Existing chronic conditions ^a^				
Vision problems	−0.044 ** (−0.073–−0.015)	−0.072 ** (−0.120–−0.024)	−0.008 (−0.027–0.010)	0.124 ** (0.042–0.207)
Hearing loss	−0.021 (−0.062–0.019)	−0.035 (−0.108–0.039)	−0.007 (−0.034–0.021)	0.062 (−0.078–0.202)
Back problems	−0.012 (−0.045–0.021)	−0.019 (−0.072–0.034)	−0.002 (−0.010–0.007)	0.033 (−0.060–0.126)
Arthritis	−0.006 (−0.039–0.028)	−0.008 (−0.060–0.043)	<0.000 (−0.004–0.004)	0.014 (−0.074–0.103)
Hypertension	−0.003 (−0.035–0.029)	−0.005 (−0.052–0.042)	<0.000 (−0.002–0.001)	0.008 (−0.071–0.088)
Heart disease	−0.030 (−0.061–0.000)	−0.051 (−0.108–0.006)	−0.012 (−0.038–0.015)	0.093 (−0.016–0.202)
Osteoporosis	0.011 (−0.037–0.059)	0.015 (−0.048–0.079)	−0.001 (−0.008–0.007)	−0.026 (−0.131–0.079)
Bladder or bowel incontinence	−0.029 (−0.073–0.015)	−0.051 (−0.140–0.039)	−0.015 (−0.068–0.038)	0.095 (−0.089–0.279)
Diabetes	−0.011 (−0.041–0.020)	−0.016 (−0.061–0.029)	<0.000 (−0.004–0.003)	0.027 (−0.050–0.104)
Obesity	−0.029 * (−0.058–0.000)	−0.048 (−0.100–0.004)	−0.009 (−0.031–0.012)	0.086 (−0.011–0.184)
None	0.017 (−0.054–0.088)	0.024 (−0.069–0.116)	−0.002 (−0.020–0.016)	−0.039 (−0.185–0.108)
Other	0.009 (−0.055–0.073)	0.012 (−0.074–0.099)	<0.000 (−0.009–0.008)	−0.021 (−0.163–0.122)
Prescription medications				
0	Reference			
1–4	0.005 (−0.044–0.055)	0.008 (−0.068–0.085)	0.001 (−0.011–0.013)	−0.014 (−0.152–0.123)
≥5	0.028 (−0.046–0.101)	0.037 (−0.060–0.135)	−0.002 (−0.019–0.015)	−0.063 (−0.233–0.106)
OTC medications				
0	Reference			
1–2	0.012 (−0.022–0.046)	0.019 (−0.032–0.069)	0.002 (−0.004–0.008)	−0.033 (−0.120–0.055)
3–4	0.005 (−0.029–0.039)	0.008 (−0.046–0.062)	0.001 (−0.006–0.009)	−0.014 (−0.109–0.081)
≥5	0.061 (−0.006–0.128)	0.074 * (0.009–0.139)	−0.017 (−0.055–0.021)	−0.118 * (−0.216–−0.020)
Received pharmacist counseling				
No	Reference			
Yes	−0.038 * (−0.071–−0.006)	−0.057 * (−0.105–−0.009)	0.002 (−0.012–0.015)	0.094 * (0.020–0.167)

Abbreviations: SAR: Saudi Arabian Riyals; OTC: over-the-counter; P-FRIDs: prescription fall-risk-increasing drugs; OTC-FRIDs: over-the-counter fall-risk-increasing drugs. ^a^ For this variable, the reference was “not having that particular chronic condition”. Asterisks represent significant group differences compared to the reference based on the ordered probit regression model and the outcome (poor knowledge, average knowledge, good knowledge): ** 0.001 ≤ *p* < 0.01; * 0.01 ≤ *p* < 0.05.

## Data Availability

Data are available upon reasonable request to the corresponding author.

## Supplementary Materials

The following supporting information can be downloaded at: https://www.mdpi.com/article/10.3390/healthcare13131549/s1, File S1: Study Questionnaire: Evaluating Fall Risk and Knowledge of fall-risk increasing drugs (FRIDs) in Saudi Community-Dwelling Older Adults. Table S1. Responses on the Stay Independent screening tool. Table S2. Knowledge assessment for prescription fall risk-increasing drugs (P-FRIDs). Table S3. Knowledge assessment for over-the-counter fall risk-increasing drugs (OTC-FRIDs). Figure S1. Relationships between number of medications and fall risk scores. Figure S2. Forest plot for adjusted odds ratios of fall risk factors. Figure S3. Comparison of knowledge levels across demographics.
