# Supplementary material for: Fall Risk and Knowledge of Fall-Risk-Increasing Drugs Among Saudi Older Adults
Source: Healthcare (Basel). 2025 Jun 29;13(13):1549. doi: 10.3390/healthcare13131549 (PMC12249347; doi:10.3390/healthcare13131549)

File S1

Study Questionnaire

Evaluating Fall Risk and Knowledge of fall-risk increasing drugs (FRIDs) in Saudi Community-Dwelling Older Adults

Greetings! You are invited to participate in a study being conducted by a research team from College of Pharmacy, Jazan University. The purpose of the study is to determine older adults' risk of fall and their current state of knowledge of prescription and OTC medications that can increase the risk of falling. This research study has been approved by the Standing Committee for Scientific Research at Jazan University, Saudi Arabia (REC-45/03/768).

Participation
Your participation in this survey is voluntary. It takes approximately 15 minutes to complete this survey. You may refuse to take part in the research or you may skip any question you do not wish to answer for any reason without any consequences.

Benefits & Risks
Participating in this survey benefits individuals and the broader community. Participants will learn about fall-risk increasing drugs (FRIDs) and their potential impact, enabling them to assess personal risk factors and take preventive measures. By contributing, participants help improve fall prevention strategies for older adults in Saudi Arabia, potentially influencing healthcare policies. Participation is cost-free, confidential, and may offer opportunities for future research. This study aligns with national health initiatives, allowing participants to impact the health and well-being of older adults in their community.

Participation in the study offers potential benefits but also carries certain risks that participants should consider. These risks include psychological discomfort arising from questions about fall risks, which may induce anxiety or stress in some individuals. Privacy concerns may persist despite confidentiality measures, as participants might worry about the security of their personal information. It is crucial to weigh these risks against the potential benefits before deciding to take part in the study.

We have implemented measures to minimize these risks. If you have any concerns, please don't hesitate to ask for clarification or support during the study.

Confidentiality
Your survey answers will be stored in a password protected electronic format. Data will later be downloaded and stored on secure servers of College of Pharmacy, Jazan University. No names or identifying information would be included in any publications or presentations based on these data, and your responses to this survey will remain confidential.

Contact
If you have questions concerning the study, contact the principal investigator by email at mhsyed@jazanu.edu.sa

**Are you at least 60 years of age or older?**	☐ YES ☐ NO
**Are you residing in Jazan province?**	☐ YES ☐ NO
**Are you a Saudi citizen?**	☐ YES ☐ NO
**Please indicate that you have read the above information.**<br>☐ *I have read and understand the previous information and agree to participate in this research study.*	

Section I: Demographics

1) **Age** ……… years
**2) Gender**<br>(1) Male<br>(2) Female
3) **Marital Status**<br>(1) Married<br>(2) Single<br>(3) Divorced/Widow
4) **Education**<br>(1) None<br>(2) Primary School<br>(3) Secondary School<br>(4) Bachelor and above
**5) Employment Status**<br>(1) Not working<br>(2) Still working
6) **Household Income**<br>(1) <5000<br>(2) 10,000 – 15,000<br>(3) 15,001 – 20,000<br>(4) >20,000
**7) Existing chronic conditions**<br>(1) Vision problems<br>(2) Hearing loss<br>(3) Back problems<br>(4) Arthritis<br>(5) Hypertension<br>(6) Heart disease<br>(7) Osteoporosis<br>(8) Bladder or bowel incontinence<br>(9) Diabetes<br>(10) Obesity<br>(11) None<br>(12) Other – If “Yes” Please specify_____________
**8) How many prescription medications are you currently taking?**<br>(1) 0<br>(2) 1-4<br>(3) ≥ 5
**9) How many over the counter medications are you currently taking?**<br>(1) 0<br>(2) 1-5<br>(3) ≥ 5
**10) In past 6 months, did you ever receive counseling from a pharmacist regarding fall risks related to medications?**<br>(1) Yes<br>(0) No

Section II: Risk of falling

Please indicate your response for each statement given below.	Yes	No
1) I have fallen in the past year.	②	⓪
2) I use or have been advised to use a cane or walker to get around safely.	②	⓪
3) Sometimes I feel unsteady when I am walking.	①	⓪
4) I steady myself by holding onto furniture when walking at home.	①	⓪
5) I am worried about falling.	①	⓪
6) I need to push with my hands to stand up from a chair.	①	⓪
7) I have some trouble stepping up onto a curb.	①	⓪
8) I often have to rush to the toilet.	①	⓪
9) I have lost some feeling in my feet.	①	⓪
10) I take medicine that sometimes makes me feel light-headed or more tired than usual.	①	⓪
11) I take medicine to help me sleep or improve my mood.	①	⓪
12) I often feel sad or depressed.	①	⓪

Section III: Knowledge Assessment of Prescription Fall Risk-Increasing Drugs

Please indicate your response for each statement given below.	Yes	No	I don't know
1) Prescription medication to help me sleep can increase my chances of falling.	①	②	③
2) Prescription medication to lower my blood pressure can increase my chances of falling.	①	②	③
3) Prescription medication to help with anxiety can increase my chances of falling.	①	②	③
4) Prescription medications (taking more than 4 medications) can increase my chances of falling.	①	②	③
5) Prescription medication for agitation can increase my chances of falling.	①	②	③
6) Prescription medication for heartburn can increase my chances of falling.	①	②	③
7) Prescription medication to improve my mood can increase my chances of falling.	①	②	③
8) Prescription medication to lower my cholesterol increase my chances of falling.	①	②	③
9) Prescription medication to help with bladder symptoms can increase my chances of falling.	①	②	③

Section IV: Knowledge Assessment of Over-the-Counter Fall Risk-Increasing Drugs

Please indicate your response for each statement given below.	Yes	No	I don't know
1) Some cold medications can increase my chances of falling	①	②	③
2) A multivitamin can increase my chances of falling	①	②	③
3) Medicines to help me sleep can increase my chances of falling	①	②	③
4) Some allergy medications can increase my chances of falling	①	②	③
5) Calcium supplements can increase my chances of falling	①	②	③

6) Panadol can increase my chances of falling	①	②	③

Section V: Willingness to discuss medication changes with your pharmacist

1. How likely would you be to discuss change or reduce your medicine if your pharmacist thought its side effects (like sleepiness, light-headedness, or dizziness) might make you fall?

(1) Very Unlikely
(2) Somewhat Unlikely
(3) Somewhat Likely
(4) Very Likely

Supplementary Table S1. Responses on the Stay Independent screening tool.

Risk of fall	Score[a]	No. (%)[b]
I have fallen in the past year.	②	151 (39)
I use or have been advised to use a cane or walker to get around safely.	②	105 (27)
Sometimes I feel unsteady when I am walking.	①	159 (41)
I steady myself by holding onto furniture when walking at home.	①	125 (32)
I am worried about falling.	①	261 (67)
I need to push with my hands to stand up from a chair.	①	170 (43)
I have some trouble stepping up onto a curb.	①	165 (42)
I often have to rush to the toilet.	①	130 (33)
I have lost some feeling in my feet.	①	104 (27)
I take medicine that sometimes makes me feel light-headed or more tired than usual.	①	87 (22)
I take medicine to help me sleep or improve my mood.	①	71 (18)
I often feel sad or depressed.	①	110 (28)

[a] According to the scale, each response as yes has a designated score. first two questions had a score of 2, and the remaining ten questions each had a score of 1. A score of four or more indicates fall risk. A negative response to any of the items received a score of zero.

[b] Percentages may not sum to 100% as participants responded "yes" or "no" to each of the items independently.

Supplementary Table S2. Knowledge assessment for prescription fall risk-increasing drugs (P-FRIDs).

Prescription medication…….. can increase my chances of falling.	No. (%)[a] Correct Answers
…...to help with bladder symptoms.	109 (27.9)
…...to lower my blood sugar.	212 (54.2)
…...to improve my mood.	205 (52.4)
…...for heartburn.	241 (61.6)
…...for agitation.	160 (40.9)
…...taking more than four medications.	193 (49.4)
…...to help with anxiety.	201 (51.4)
…...to lower my blood pressure.	222 (56.8)
…...to help me sleep.	316 (80.8)

[a] Percentages may not sum to 100% as participants responded "yes", "no" or “I do not know” to each of the items independently. Each correct answer received a score of one and a wrong answer or “I do not know” response was scored as zero. The total number of correct answers for each respondent were added and characterized into knowledge levels based on the percentage of correct answers (> 80% correct answers indicating good knowledge, 60-80% signifying average knowledge, and < 60% denoting poor knowledge).

Supplementary Table S3. Knowledge assessment for over-the-counter fall risk-increasing drugs (OTC-FRIDs).

Questions pertaining to OTC medications with fall risk	No. (%)[a] Correct Answers
Panadol can increase my chances of falling.[b]	265 (67.8)
Calcium supplements can increase my chances of falling.[b]	292 (74.7)
Some allergy medications can increase my chances of falling.	124 (31.7)
Medicines to help me sleep can increase my chances of falling.	255 (65.2)
A multivitamin can increase my chances of falling.[b]	266 (68.0)
Some cold medications can increase my chances of falling.	156 (39.9)

[a] Percentages may not sum to 100% as participants responded "yes", "no" or “I do not know” to each of the items independently. Each correct answer received a score of one and a wrong answer or “I do not know” response was scored as zero. The total number of correct answers for each respondent were added and characterized into knowledge levels based on the percentage of correct answers (> 80% correct answers indicating good knowledge, 60-80% signifying average knowledge, and < 60% denoting poor knowledge).

[b] Indicates a negative question

Supplementary Figure S1 – Comparison of knowledge levels across demographics

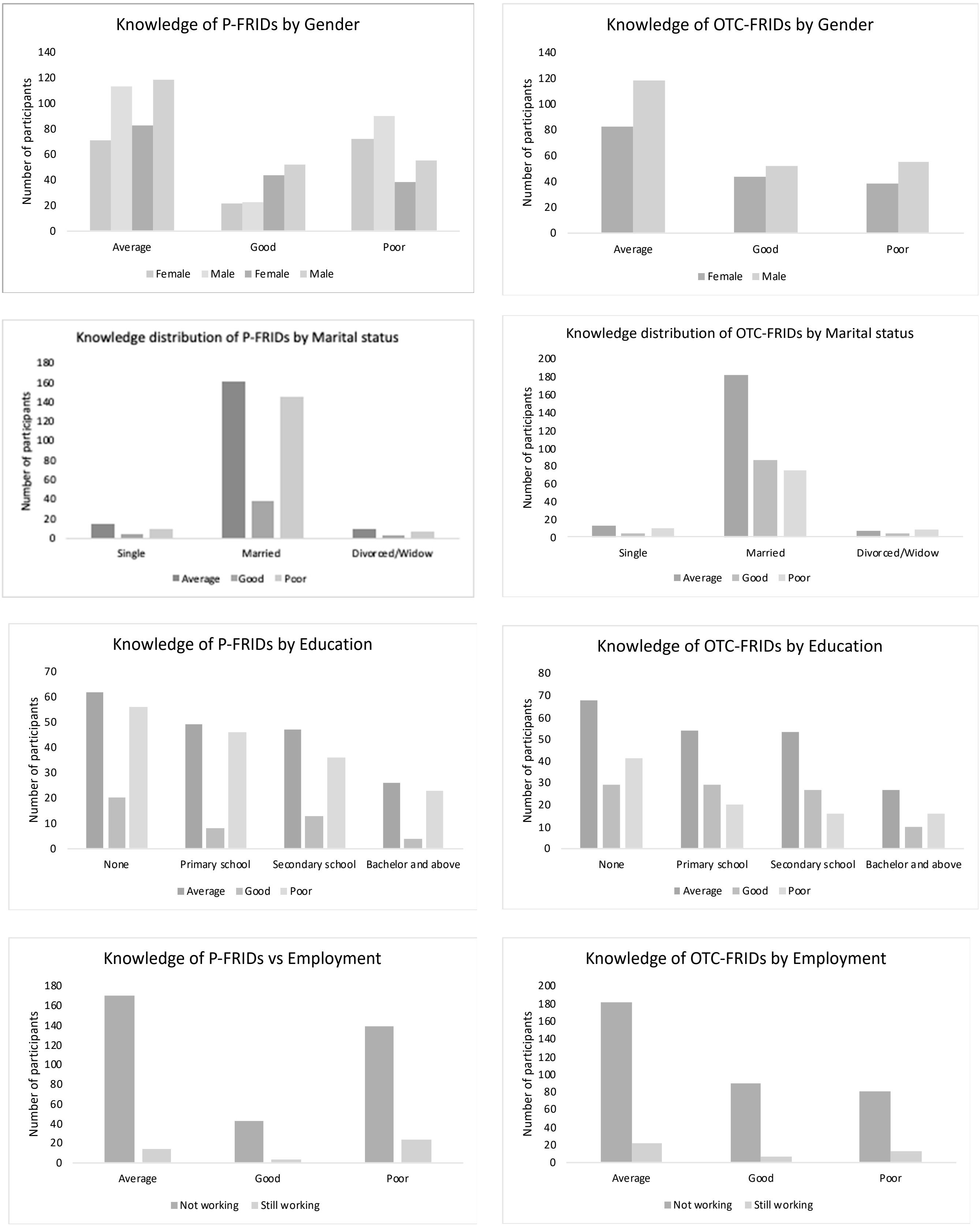

Supplementary Figure S2 – Relationships between number of medications and fall risk scores

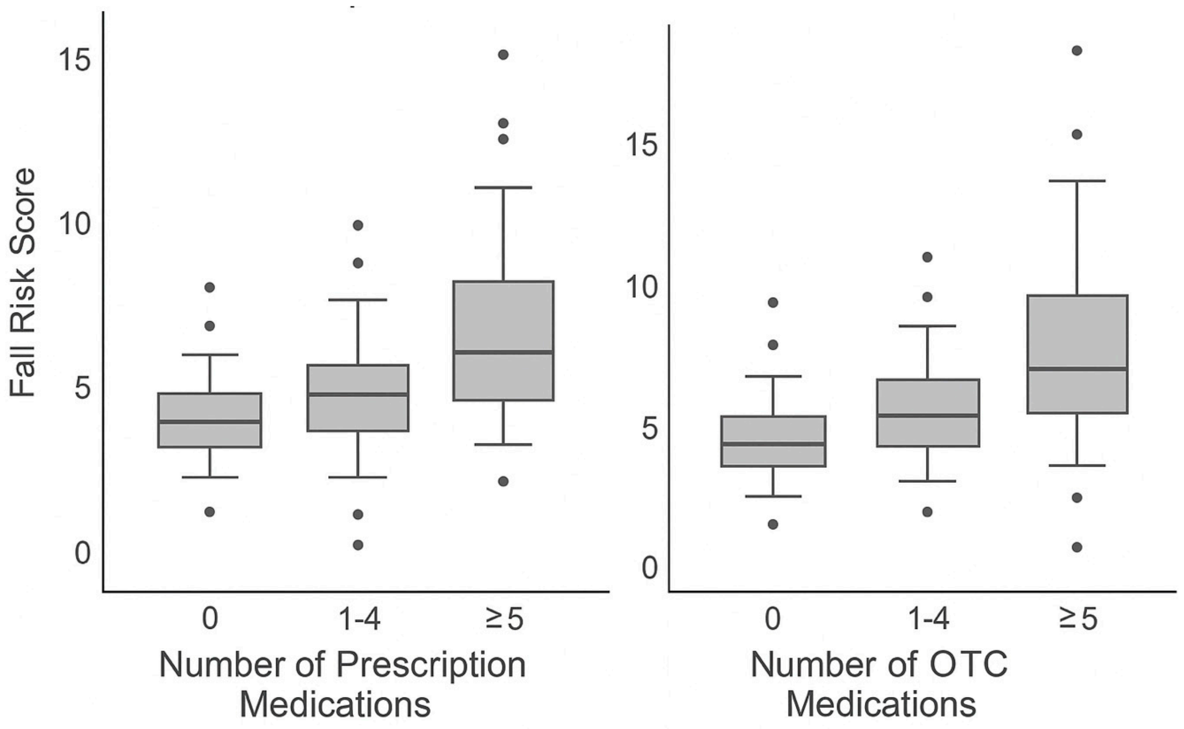

Supplementary Figure S3 - Forest plot for adjusted odds ratios of fall risk factors

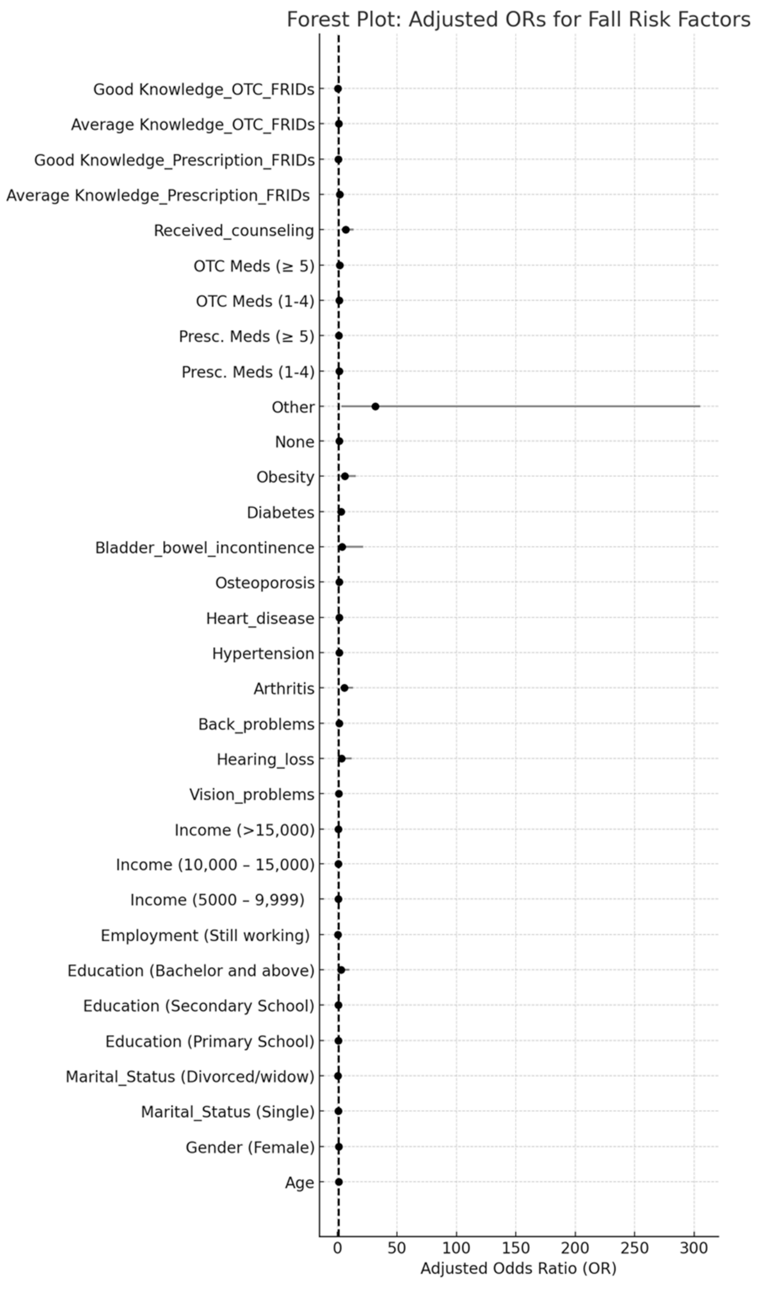

Supplement: Supplementary file 1 [file healthcare-13-01549-s001.zip › healthcare-3633590-supplementary.pdf]
